# Epidemiological geography at work: An exploratory review about the overall findings of spatial analysis applied to the study of CoViD-19 propagation along the first pandemic year

**DOI:** 10.1007/s10708-022-10601-y

**Published:** 2022-03-29

**Authors:** Andrea Marco Raffaele Pranzo, Elena Dai Prà, Angelo Besana

**Affiliations:** 1 Geo-Cartographic Centre for Studies and Documentation, University of Trento, Trento, Italy; 2 Interuniversity Department of Regional and Urban Studies and Planning, Polytechnic of Turin, Torino, Italy

**Keywords:** COVID-19, GIS, Health Geography, Medical Geography, Spatial analysis, Pandemic determinants

## Abstract

The present work aims to give an overview on the international scientific papers related to the territorial spreading of SARS-CoV-2, with a specific focus upon applied quantitative geography and territorial analysis, to define a general structure for epidemiological geography research. The target publications were based on GIS spatial analysis, both in the sense of topological analysis and descriptive statistics or *lato *sensu geographical approaches. The first basic purpose was to organize and enhance the vast knowledge developments generated hitherto by the first pandemic that was studied “on-the-fly” all over the world. The consequent target was to investigate to what extent researchers in geography were able to draw scientifically consistent conclusions about the pandemic evolution, as well as whether wider generalizations could be reasonably claimed. This implied an analysis and a comparison of their findings. Finally, we tested what geographic approaches can say about the pandemic and whether a reliable spatial analysis routine for mapping infectious diseases could be extrapolated. We selected papers proposed for publication during 2020 and 209 articles complied with our parameters of query. The articles were divided in seven categories to enhance existing commonalities. In some cases, converging conclusions were extracted, and generalizations were derived. In other cases, contrasting or inconsistent findings were found, and possible explanations were provided. From the results of our survey, we extrapolated a routine for the production of epidemiological geography analyses, we highlighted the different steps of investigation that were attained, and we underlined the most critical nodes of the methodology. Our findings may help to point out what are the most critical conceptual challenges of epidemiological mapping, and where it might improve to engender informed conclusions and aware outcomes.

## Introduction

### Background

Pandemics are multifactorial events with complex origin (Che, 2019), and many of their contributing determinants, both fostering or hampering the infection, are not well-known, or even unknown (Tscherne and García-sastre, 2011). Territory represents the logical framework where epidemiological determinants’ influence can be traced (Eisenberg et al., 2007), but also, pragmatically, the field where their assessment becomes partially possible (Kundi, 2006). However, the mere territorial coincidence being not decisive to prove causality nexuses (Susser, 1991), it becomes imperative to set out strong interdisciplinary methodologies to attain consistent validations whereas geographical factors are explicitly invoked as amplifying or hindering elements for the pandemic propagation, to avert confounding effects (Vineis, 2003). Alongside, the use of cartography and GIS in infectious disease studies has traditionally been a strong line of applied research, both in the sense of providing the professionals with the technical tools able to systematize epidemiological information within a geographical framework apt to inform the management of disease control, and in the sense of summarizing scientifically based information to be communicated to the general public (Carroll et al., 2014). Nonetheless, quantitative geography brings with it a series of specificities which imply a high level of awareness about the potential and the limitations of cartography as an epidemiological instrument.

### Context

During 2020, the researchers had to set out the systematic analysis of a partially unknown phenomenon while it was and is still (quickly) evolving day by day. In hindsight, the geographic research carried out during the first year of the pandemic developed within two different contexts. The first pulse of the pandemic, approximatively identifiable with the “2020 spring wave” (from the point of view of the temperate latitudes of northern hemisphere) forced the researchers to react to an almost unknown emergency that was spreading at a fast pace across mankind. During this early stage, the virus was substantially able to propagate freely in a business-as-usual scenario. Accordingly, geographers had the opportunity to sketch out an inventory of the epidemiological dynamics within several human networks. GIS became a “radioactive tracer” potentially able to spot the “basin outlets” through which the infection was exported from one community to another, thus giving a bird’s eye view on what was happening in each territorial subset of mankind. During the second stage, also mediatically known as “second wave” (2020 autumn pulse), the context radically changed. After the failure of containment measures, mitigation interventions were massively implemented, together with an increased preparedness of the medical staff, and the virus propagation restarted its exponential behavior from endemic sources, spreading not in the absence of countermeasures, but notwithstanding them. Hence, geographers were supposed to partially readjust their perspective and to confront local variances in the pandemic propagation, whose anisotropy was deemed to mirror—more or less faithfully—unconformities in the anthropic fabric of human collectivities. Our approach was, therefore, to identify the most developed investigation pathways that epidemiological geography began to tread in the quantitative study of the COVID-19 pandemic. Clearly, such a work could not be in any case considered exhaustive. The narrower purpose was to gather a selection of sources and offer an organic summary apt to build further research perspectives. The second scope was to discover to what extent geographers and researchers were able to find and explain recognizable patterns in the pandemic spreading, and to check if any shared commonalities could be excerpted. The third objective was to look for the existence of a routine in epidemiological geography able to produce reliable scientific information, and if so, to understand which methodology could be derived. Overall, this survey provided a hint about the urgency of an increased methodological awareness while addressing the geography of infectious diseases, because we deem that GIS could be a pivotal element in this subject (Charlier et al., 2020), but under the strict condition of a careful check of the classic biases of quantitative geography, as well as the full acknowledgement of the intrinsic interdisciplinarity of this field.

## Methods

To explore and sift the highest possible number of spatial correlation tests and pandemic pattern studies, an iterative “browsing and berrypicking” process was carried out, as suggested by a classic online exploratory research approach (Bates, 1989). A series of specific keywords was applied in combination with Boolean operators, resumable with the synthetic expression *“[#GIS AND (#Geography OR #Cartography OR #Mapping OR (#Spatial AND #Analysis))] AND [#COVID OR #Coronavirus OR #SARS-COV-2]*”, to investigate six search engines (*Google Scholar*, *Scopus*, *DOAJ, IURN, CORE, BASE*) and the periodical reports issued by the *Institute of Development Studies* (*K4D COVID-19*). Finally, the sole two content reviews found hitherto (Ahasan et al., 2020; Franch-Pardo et al., 2020) were addressed as a benchmark to validate the query outcome. It was deemed acceptable to overlook a well-known inconvenient (Gusenbauer, 2019) concerning the fact that the exact repeatability of a single Boolean query is not guaranteed even in the short term, independently from the natural growth of online repositories. The queries were performed between July 1st and July 8th,2020 and between November 25th and December 15th,2020. Two parameters were defined to filter the relevant papers: 1) The study has a geographical-epidemiological perspective or design; 2) the study uses spatial/geostatistical analysis, GIS, or cartography in its rationale, and considers the territory as an element of inquiry. As the present survey was merely explorative, several papers that had not yet undergone the peer-review process were also included, by marking them with the acronymous NPR (*not peer-reviewed*) or APP (*Advanced Preprint Publication*) or PP (*Pre-Print*) in the table. Likewise, some *Working Papers* (WP) were included. The papers were assessed by considering their study area, their scale ratio, the prevailing type of data, the methodology—divided, wherever possible, in general statistic methods and more specifically geostatistical or GIS methods—and their findings. Through this routine we sifted, screened and classified the geographic analyses with the scope to identify common research patterns, shared methods, generalizable findings and cross-cutting biases.

## Results

### Raw data: general overview

**Fig. 1:**
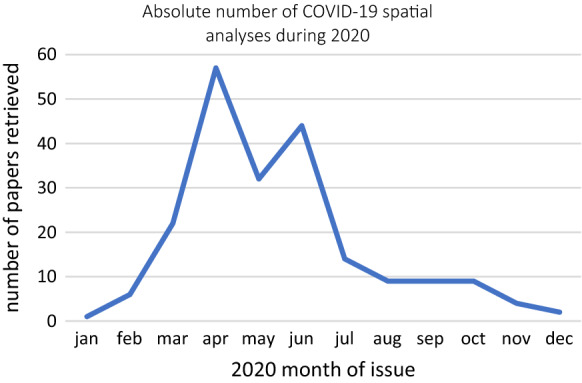
The surveyed papers divided by month of publication

The results are available at https://doi.org/10.5281/zenodo.4685964. According to the above-mentioned criteria, 209 papers were selected to undergo the survey. The trend in absolute number of spatial analyses devoted to COVID-19 geographical studies is shown in Fig. 1. Indeed, the pace of production of these studies could have been heavily altered by the lockdown affecting the researchers’ activities, and, in late 2020 by the reintroduction of classic peer-review processes after a first period of pre-print circulation allowed by many journals.

**Fig. 2:**
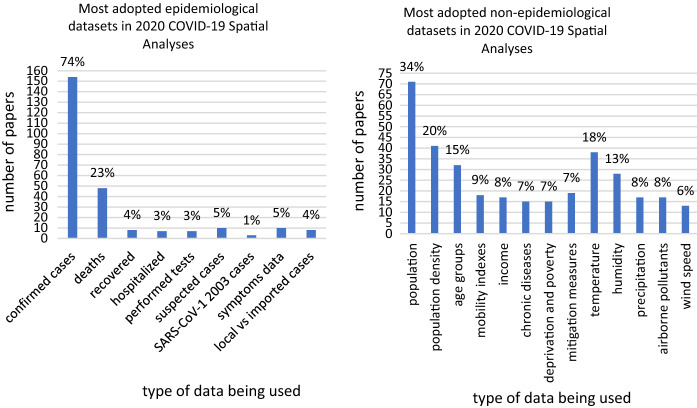
On the left (**a**) the surveyed papers sorted by the epidemiological datasets they adopted. On the right (**b**), the surveyed papers sorted by the non-epidemiological datasets they used to test the existence of correlations

Among the 209 output papers that complied with the selection criteria, 154 papers (≈74%) relied upon daily and/or cumulative cases for their conclusions, 48 (≈23%) extended their analysis to deceases, 8 (≈4%) to recovered, 7 (≈3%) to hospitalized, 7 (≈3%) to performed tests. In 10 cases (≈5%) the authors had to rely upon suspected cases. Only 3 (≈1%) papers attempted to make comparisons with 2003 SARS-CoV-1 confirmed cases. 10 papers (≈5%) utilized symptom-related data, and 8 (≈4%) dealt with the disaggregation of local infections and imported cases. Albeit being one of the most precious datasets, the usage of the latter was significantly lower than expected, probably because of the meteoric fading of local health authorities’ ability to keep recording this differentiation within the frantic course of the first wave of infections, and maybe, we suppose, also due to the early decision of almost all countries to give up the containment and focus only on mitigation (Fig. 2a).

**Fig. 3:**
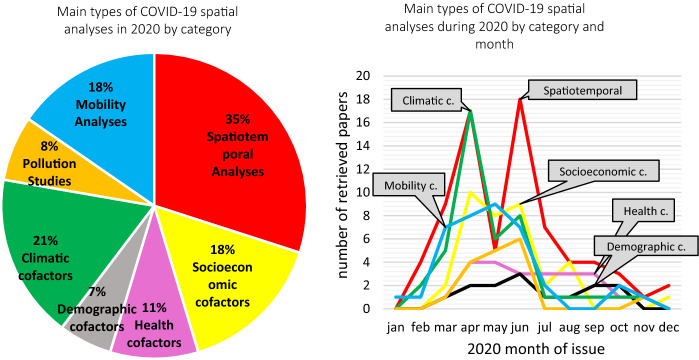
The surveyed papers divided by thematic category (left, **a**) and by month of publication (right, **b**). 27 studies were inserted in more than one single category

Moving to the non-epidemiological data processed (Fig. 2b), as expected, population was the most used type of data (71 papers, ≈ 34%), followed by population density (41 papers, ≈20%), age groups (32 papers, ≈15%), mobility indexes (18 papers, ≈9%), income (17 papers, ≈8%) chronic diseases (15 papers, ≈7%), and deprivation and poverty (15 papers, ≈7%). At least 19 papers (≈9%) also considered mitigation measures and their date of enforcement. As for the environmental data, temperature was the most investigated variable (38 papers, ≈18%), followed by humidity (28 papers, ≈13%), precipitation (17 papers, ≈8%), airborne pollutant concentration (17 papers, ≈8%), and wind speed (13 papers, ≈6%). The researchers, overall, addressed the main traditional epidemiologically relevant independent variables in quite different proportions. Verisimilarly, their choice was often driven by data availability, with a predictable preference for demographic data and far lower percentages for crucial factors that can be less easily retrieved (e.g., local trends for chronic diseases) or less effectively built (e.g., indicators on human mobility). Demographic data, together with the temporal dimension, were the promptest solution to give a first insight to the health emergency and they were used to build up a huge number of spatiotemporal analyses during the first pandemic wave (Fig. 3b) and its doubled pulse (the Chinese primary outbreak and then the secondary major outbreaks in Europe, the US, Brazil, India). A parallel peak could be noticed for the use of environmental and climatic data: temporally, this choice could be related to the strong pressure deriving from the authorities and the general public in knowing whether the pandemic would be faded by the arrival of boreal summer, or whether the virus propagation would be hindered within tropical climate ranges (Fig. 3b).

On the basis of our thematic classification (Fig. s 3a and 3b), 74 (≈35%) papers were presented in the form of spatiotemporal analyses, while 38 (≈18%) analyzed the socioeconomic context of the pandemic. 23 papers (≈11%) considered preexisting health factors, and 14 (≈7%) the demographic structure. Environmental factors were studied by 43 papers (≈21%), while pollution by 17 papers (≈8%). Mobility was the main subject of 38 papers (≈18%). 27 out of 209 papers were included in more than one category (≈13%). A specific cross-cutting analysis is provided for each category in Paragraph 3.2, to understand the stronger or weaker explanatory efficacy reached within each subject, without detracting from the assumption that in an idiographic discipline it is usually inadvisable to generalize single case studies.

**Fig. 4:**
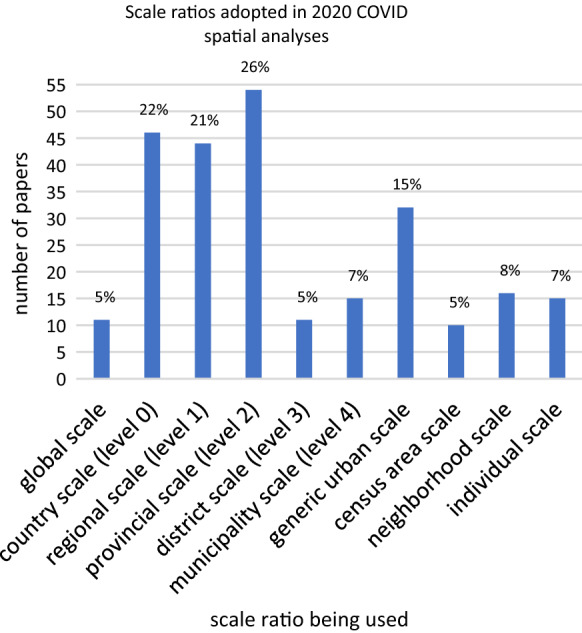
The surveyed papers divided by the scale ratio they adopted. Please note that the various administrative subdivisions were roughly grouped and homogenized based on their placement within each national administrative hierarchy, and the English names are just for indicative purposes (e.g., the term “Province” in China is usually used for level 1, while in Italy for level 2; in federal countries federate states correspond to level 1, while in non-federal countries usually regions correspond to level 1; and so on)

Data availability and their level of disaggregation may also explain the peculiar distribution of the scale ratio chosen for the analyses (Fig. 4). 11 studies (≈5%) adopted a global scale ratio, while, amongst the papers that investigated the pandemic at the subnational scale, 32 (≈15%) analyses adopted a generic urban scale, but only 15 (≈7%) a formal municipality scale. 16 (≈8%) papers used the neighborhood scale, 10 (≈5%) the census area scale and 15 (≈7%) the individual scale. Unsurprisingly, the country scale was among the most recurring options (46, ≈22%), as the most easily available open datasets are generally gathered and released by national health authorities or are aggregated by country by the biggest data aggregators. However, a similar or even higher number of analyses was carried on at the regional (44) and provincial (54) scale, thus revealing that many research teams had at least a fair accessibility to intermediate scale datasets. Less intuitively, analyses at the finest geographical scale ratios (neighborhood, census area) were not markedly more frequent than the individual scale ratio: this could be due to the combined effect of the severe data scarcity at a very granular level, and the increasing diffusion of studies based upon mobile phone tracking data.

**Fig. 5:**
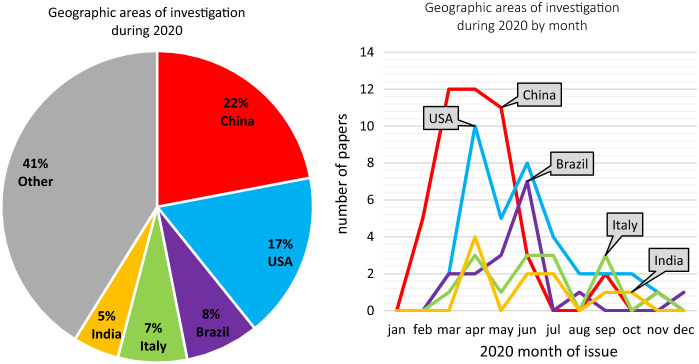
The surveyed papers divided by country of investigation (left, **a**) and by month of publication (right, **b**). Only the first five most recurring countries are disaggregated. Red: China. Light blue: USA. Purple: Brazil. Green: Italy. Orange: India

The most studied countries (Fig. 5a) were China (47 papers, ≈23%), the U.S. (36, ≈17%), Brazil (16, ≈8%), Italy (15, ≈7%) and India (10, ≈5%). Taking into account the ordinary delay due to data processing, writing and peer-review validation, it is easy to check out (Fig. 5b) how the choice upon the case study areas was strongly determined by the concrete pattern of the pandemic. Concomitantly with the very first pandemic pulse (January 2020), the highest proportion of publications used China (and mainly Hubei province) as their study area (March/April 2020). A second possible concentration was in June, when a second “peak” can be seen relatively to papers analyzing Brazil and the United States, which experienced a catastrophic exponential growth in infections along the previous month. A smoother research focus might be represented by the studies investigating two other severe nationwide outbreaks: Italy and India. These first observations may witness to what extent the evolution of the object of study did affect the researchers’ approaches and their study designs along the whole period, and how that influence was remarkable even upon basic elements like the scale ratio, the studied area, and the supplementary data, thus conditioning the category of spatial analysis that each research team finally chose.

**Fig. 6:**
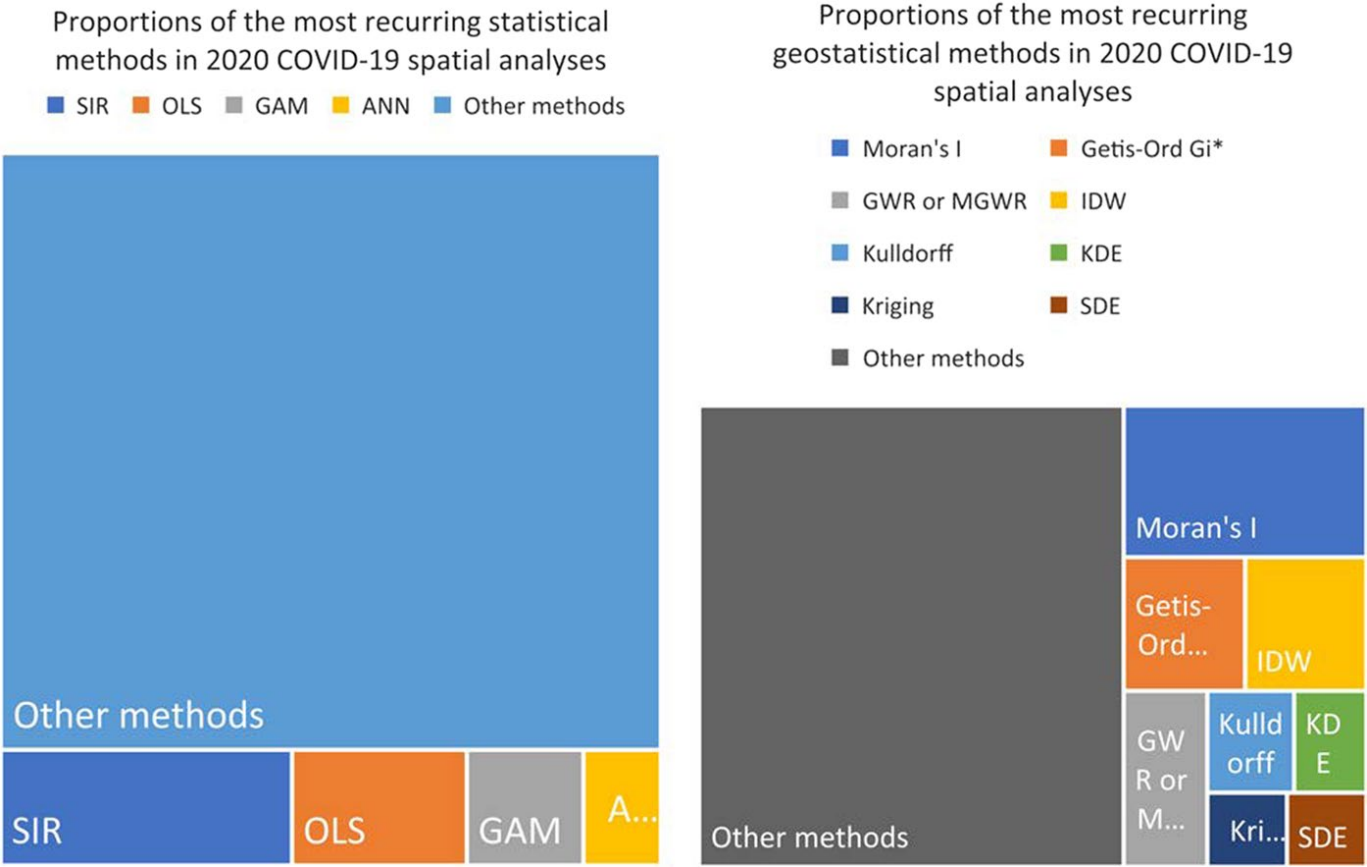
The surveyed papers divided proportionally by the statistical methods they employed (left, **a**) and by the geostastical methods they employed (right, **b**). Only the first most recurring methods are disaggregated out of the total

As for the methodology (Fig. 6a), the statistical approaches were frequently based upon by the adoption of the classic epidemiological deterministic compartment models like SIR (Susceptible—Infected—Recovered) and its modified versions, including the stochastic ones, to treat the datasets (15 papers, ≈7%). Other slightly recurring methods were the ordinary least square regression OLS (9 papers, ≈4%) the generalized additive method GAM (6 papers, ≈3%) and the artificial neural network (ANN) -based models (4 papers, ≈2%).

Furthermore, some recurring geostatistical methods were detected (Fig. 6b): 25 papers (≈12%) relied upon the Moran’s I spatial autocorrelation index, whose 14 papers (≈7%) explicitly addressed its local version based upon Anselin’s LISA method, while 11 papers (≈5%) carried out hotspot analysis through the Getis-Ord Gi* statistic. Among the several existing regression techniques, 10 papers (≈5%) opted for the geographically weighted regression (GWR) often in its multivariate version (MGWR). 11 papers (≈5%) adopted the inverse distance weighting (IDW); 6 papers (≈3%) applied the Kulldorff’s Poisson prospective (or retrospective) spatial-scan statistic. Other less recurring spatial statistics were the kernel density estimation, KDE (5 papers, ≈2%), the kriging interpolation and its modified versions (4 papers, ≈2%), and the standard deviation ellipse SDE (4 papers, ≈2%). We will further discuss a possible way to frame this variegated array of methods in Paragraph 3.3.

As for the findings of the surveyed papers, we grouped them in seven thematic categories, so that their contents could be *lato *sensu comparable:

3.2.1 Spatiotemporal analyses

3.2.2 Studies on socioeconomic cofactors

3.2.3 Studies on health cofactors

3.2.4 Studies on demographic cofactors

3.2.5 Studies on environmental and climatic cofactors

3.2.6 Studies on pollution

3.2.7 Mobility analyses

### Categorized data: in-depth screening

#### Spatiotemporal analyses

This category encompasses the papers that primarily focused upon the very geographical characteristic of the pandemic, i.e., its territorial propagation with the passing of time. Spatiotemporal analyses were the most frequent and multidisciplinary contributions. They tried to answer a simple but paramount question: “what did happen within a specific area?”. Research strategies were quite diverse, but all built on the widely verified assumption that pandemic data showed strong spatial autocorrelation (Ye and Hu, 2020) and that spatial autoregressive models could approximate the real behavior of the pandemic better than aspatial models (Sun et al., 2020).

From a more “nomothetic” point of view, it was possible to draw out some tentative generalizations. Among the 74 surveyed spatiotemporal analyses, 15 (≈20%) found that confirmed cases were spatially autocorrelated; 5 (≈7%) found that the local propagation rate of the pandemic was positively correlated with the proximity to a known outbreak; 5 (≈7%) found that the insurgence of secondary outbreaks was positively correlated to the proximity to a primary outbreak; 9 (≈12%) found that the insurgence of secondary outbreaks was positively correlated to the level of connectedness between the area of insurgence and the area affected by a primary outbreak; 12 (16%) observed that the mitigation measures had a measurable impact upon the spreading of the virus. 10 (14%) observed that at the local level, the pandemic attacked a given society starting from the wealthier neighborhoods and then propagated across the poorer areas; 5 (≈7%) noticed that the apparent primary hotspot in a given area was not the very first outbreak of the given community; 5 (≈7%) observed that, at any scale ratio, the spreading of the pandemic was sustained by the occurrence of recognizable superspreading events.

Altogether, 11 papers (≈15%) were able to deal with at least some medically ascertained epidemiological links. 13 papers (≈18%) directly engaged the time dependency of the COVID-19 dynamics. 9 papers produced prospective scenarios or simulations, and 26 papers (≈35%) carried on the primary mapping of the epidemiological situation of a given area for the first time.

The very first spatiotemporal studies tried to extract new information about the extent of the pandemic from geographical patterns. Many authors noticed that, during the early stage of the pandemic, in each Chinese province the confirmed case number was positively correlated to the intensity of travelers’ flows incoming from Wuhan or Hubei (Tang et al. 2020); however, in the following stage this correlation vanished, and new secondary outbreaks occurred. The strict Hubei lockdown led finally to the exhaustion of the community transmission (Fan et al., 2020; Ye and Hu, 2020). Sun et al. (2020), merging the knowledge about coronaviruses’ phylogenetics, potential animal carriers and anthropic disturbance of their distribution, framed the conditions that could have fostered the SARS-CoV-2 cross-species transmission.

Several authors dealt with the relationship between primary and secondary outbreaks: by disaggregating the growing infection data, Huang et al., (2020, China, PP) pointed out that the second massive Chinese COVID-19 outbreak was located in Wenzhou city, while Arab-Mazar et al., (2020) observed that, albeit Teheran had got the highest number of confirmed cases, Qom should be indicated as the Iranian primary outbreak. Desjardins et al. (2020, USA) proposed a mapping method that allowed emerging COVID-19 clusters detection: the early clusters were related to King County (Washington) and Westchester County (New York). During the same weeks, the first geographical works about intertropical countries were published. The general feeling of the researchers was that they had to face a critical lack of epidemiological data, or an inefficient local virus transmission, or both conditions (Likassa, 2020). Nevertheless, Adekunle et al. (2020) managed to extract a linear relation between confirmed cases and suspected COVID-19 deaths throughout 52 African countries, while Mousavi et al. (2020) ascertained that in Afghanistan, after an infected pilgrim’s return from Qom (Iran), almost all provinces were infected. In India, the researchers observed that, even if the first cases were in Kerala (three students back from Wuhan), the first community transmission epicenter occurred in Maharashtra state (Bag et al., 2020).

In the Americas, Melin et al. (2020) drafted the phases through which the pandemic took root in Mexican states; Zambrano et al. (2020, Honduras, NPR) noticed that emerging positive cases seemed to come mainly from the biggest urban settlements; Cuartas et al., (2020) studied the infection pattern in the Colombian city of Cali, while in Brazil Santos et al. (2020) did the same for Rio de Janeiro. Still at the urban scale, but halfway around the world, Kalabikhina and Panin (2020, Russia) estimated the virus propagation speed across the metropolitan area of Moscow.

Afterward, with the exponential and ubiquitous growth of confirmed cases, the researchers performed deeper geostatistical analyses on the massive amount of data. Bermudi et al., (2020, NPR), while studying the pandemic effects in São Paulo, observed the shift of epidemic risk from the more affluent neighborhoods to the deprived ones. This detail was pointed out also by other urban-scale studies in the Americas (Cuartas et al., 2020, Colombia; Santos et al., 2020, Brazil; Zhang and Schwartz, 2020, USA).

Kergaßner et al. (2020, NPR) simulated the three-phase spatiotemporal behavior—from international case import, to local superspreading events, to exponential growth—through which the pandemic aggressed Germany, and noticed that the southwestern federate states were the most affected; likewise, Scarpone et al. (2020) identified a strong North–south COVID-19 incidence rate gradient, and warned that many examined independent variables tended to exhibit multiple non-linear behaviors. Some spatiotemporal analyses also shared a specific target, the *ex-post* assessment of mitigation effects, and stated unanimously that the government interventions, whereas timely adopted, were concretely able to modify the community transmission (Fan et al., 2020).

#### Studies on socioeconomic cofactors

Contemporary societies exhibit a high internal diversification, and socioeconomic factors are expected to be relevant in driving the infectious dynamics (Khalatbari-Soltani et al., 2020), as already perceivable from the first retrospective cohort studies (Shi et al., 2020). Geographic analyses in this category attempted to shape the possible interactions between the pandemic and the substratum of socioeconomic heterogeneity of the susceptible population, with the aim to understand whether preexistent inequalities could mirror inequalities in terms of epidemiological outcomes.

Overall, among the 38 available studies on socioeconomic cofactors, 11 papers (30%) had a specific a priori approach and produced one or more vulnerability maps or risk maps taking into account many of the variables that are going be mentioned below. Among the a posteriori approaches, 12 papers (≈32%) found relevant association between the pandemic spreading and population density, and 4 papers (≈10%) found relevant association between the pandemic spreading and absolute population entity. 13 papers (≈34%) found relevant association between COVID-19 incidence and household financial status. In particular, deprivation (4 papers), income inequality (5 papers) and income/GDP per capita (6 papers) were found to be associated to the pandemic spreading. 5 papers (≈13%) found relevant association with the usage of private/public transport, and 5 papers (≈13%) found relevant association with the urban/rural classification of the given administrative units. 6 papers (≈16%) found relevant association with local job market features (job type, essential services, low-income jobs, unemployment) and 3 papers (≈8%) with the level of education. 7 other papers (≈18%) found that social connectedness and social interaction were also significantly associated with the propagation of the pandemic, and 3 papers (≈8%) that the family structure and the typology of dwelling had significant association with the infections. More widely, 6 papers (≈16%) found that the granular features of the built environment (presence, distance and density of shops, supermarkets, bus/subway stops, road network etc.) were statistically associated to the pandemic evolution across urban populations.

As expected, population density was the main investigated covariate, and it gave the strongest signal of statistical association with the pandemic spreading. Human community uneven distribution across the countries can therefore be claimed as the most general and immediate anisotropic factor regarding the asymmetrical territorial propagation of COVID-19. Other relevant covariates may follow. First, socioeconomic indicators like income, poverty, deprivation—when related to financial status at the household scale—were often found to be correlated to the pandemic dynamics. These indicators, however, did not manifest unidirectional forms of covariance: the wealthier or more deprived condition of a given urban area resulted, from time to time, in a hindering or boosting cofactor. Admittedly, the inequality—and not the absolute degree—in economic wellbeing across the communities was a good predictor of pandemic outcomes at the local level, as it can emerge within a comparative overview, and as it is indeed a secondary proxy of the primary epidemiological determinant related to the “host’s condition”. Secondly, another relevant covariate was traced in the “built environment”, a wide definition through which the researchers tried to find a connection between the physical structure of highly urbanized communities and the COVID-19 human-to-human transmission. In this case, again, the location, travel-time, density of facilities like shops, supermarkets, public transports were used as a secondary proxy for the classic epidemiological determinant related to the “host’s behavior” but also to the “host’s environment”, as the built environment tacitly determines humans’ displacements, points of gathering and occasions of diminishing the interpersonal distance below the threshold apt for an effective airborne viral transmission. It is obviously a “statistical space” where interactions are multiple and non-linear and where many uncontrollable variables have to be parametrized, but it did not prevent some researchers to draft risk maps (e.g., Sangiorgio and Parisi, 2020) or to apply the ecological niche model in order to forecast the possible future urban hotspots (e.g., Ren et al., 2020) or just producing feasibility studies for the implementation of social distancing in crowded suburbs with vector analysis (Gibson and Rush, 2020).

This category is highly heterogeneous, but a general trend can be noticed, as the research questions were mainly built up in the form of exploration of spatial correlations between variables. A common thread was economic inequality. In the United States, this issue led inevitably to the profound and unsolved ethnicity-based injustices, which reverberate on the healthcare service access. Mollalo et al. (2020), by investigating the U.S. at the county scale, found significant association between infections and income inequalities, urban deprivation, crowding and ethnic-economic segregation. Conclusions were consistent in several contributions: minority status, low level of English, family structure, private transports, type of dwelling and disability were good predictors of local cumulative cases (Karaye and Homey, 2020). Moreover, while cold-spots were more likely to be found in affluent neighborhoods, where levels of education, percentage of white people and proportion of managers are greater, hotspots tended conversely to be found in areas with lower education levels, larger households and higher percentage of African-American inhabitants (Maroko et al., 2020), and that happened even despite population density patterns.

Besides, the different behavior of urban and rural environments emerged: Ramirez and Lee (2020, USA), observed that not only some factors seem to be associated with high incidence rates, as population density and asthma prevalence (in urban areas) and poverty and unemployment (in rural areas), but also that, while absolute death percentage was higher in urban counties, case-fatality rate was higher in rural counties. Also, in France, Amdaoud et al., (2020) found positive correlation between infections and income inequalities, population density and percentage of workers in essential services.

Shortly thereafter, the situation in Germany was investigated by Scarpone et al. (2020); the researchers concluded that built-environment density and socioeconomic variables were important predictors of incidence rates, however, the strongest factors were community interconnectedness, geographic position, transport networks and job-market structure.

Studies about the Chinese situation led to similar conclusions: in Wuhan COVID-19 prevalence appeared positively correlated to population density, urbanized soil, tertiary value per area, good sales per area, and negatively correlated to mean building dimension, PIL per area and hospital density (You et al., 2020). Throughout China, the infection trends by county showed an extremely strong correlation with resident population, mobility index, local GDP, and good sales, however the association with population density appeared to be strong only at the county level, not for the prefectures (Xiong et al., 2020), thus revealing unexpected scale-dependent correlation patterns.

#### Studies on health cofactors

Studies on health cofactors endeavored to answer this question: “to what extent the pre-existing sanitary situation could have an impact on the general outcomes of the pandemic in a given territory?” On this basis, at least two main research lines were identified: retrospective works, that explored how chronic conditions already affecting a community could modify the trend of epidemic indicators, and prospective works, that attempted to map the organization of the health systems to understand which patient burden and which kind of cure could be sustained before trespassing critical thresholds of collapse, thresholds that Gross et al., (2020) statistically detected in the detachment of death and recovery rates from new infection number decay during the lockdown.

Once the statistical advantage that an increased number of trained personnel have in mitigating COVID-19 mortality was clarified, many authors investigated the health system capacity to sustain the pandemic impact (Verhagen et al., 2020, UK; Silalahi et al., 2020, Indonesia). Besides medical resource scarcity (Zhou et al., 2020, China), most studied predictors for the shaping of the health demand surge were elderly population, education, unemployment, exposure to poverty and deprivation, and interesting suggestions about the cartographic quantification of sanitary burden were proposed at suburban scale to ease adjustments (Verhagen et al., 2020, UK; Ogojiaku et al., 2020, USA). Health service accessibility was a recurrent issue: by applying big-data raster analysis, some continent-wide studies were carried out to compare the healthcare venue availability (Geldsetzer et al., 2020). Finally, a group of papers analyzed the role of chronic conditions and their geographic prevalence in affecting the pandemic trends, treating them as a predictive risk factor (Melin et al., 2020, Mexico), or seeking statistical correlations at the local level (Ramirez and Lee, 2020, USA), or in retrospective cohort studies (Zambrano et al., 2020, Honduras, NPR).

Overall, 9 out of 23 papers of this category (≈39%) addressed the study of the influence of pre-existing chronic diseases on the outcome of the pandemic propagation; 3 out of them found relevant spatial association between chronic disease clusters and COVID-19 clusters. Other 5 utilized the pre-existing chronic disease patterns to extract vulnerability maps and vulnerability indexes. The most investigated chronic diseases were diabetes (6), heart diseases (5), hypertension (3), asthma (3), obesity (3); also, incidence rates of pneumonia, cancer, suicide, overdose, disability, depression, smokers, mental illness, and HIV were taken in consideration for correlation tests with COVID-19 incidence rate and above all with case-fatality rate.

It may be surprising that the spatial correlation between chronic conditions was investigated (and found) in quite few cases, since the correlative nexus between the two variables was soon robustly ascertained at the individual level (Liu et al., 2020). A possible explanation could be that, collectively, this kind of correlation may easily fade whereas many parallel confounding factors can alter the statistical outcome of the disease, like the socioeconomic factors mentioned hereinabove.

As for the logistic studies, 6 papers used the number of hospitals as a spatial proxy, 5 papers utilized the availability of hospital beds, and 5 papers the availability of ICU beds. 7 papers (≈30%) addressed the risk of sanitary resource scarcity, including medical personnel and nurses. The threat of hospital saturation was the core of 6 analyses (≈26%), which intended to offer practical indications about the most fragile sanitary districts and the geographical areas which were more prone to health system collapse.

#### Studies on demographic cofactors

The pandemic propagation was supposed to be strongly correlated with the population composition (Beam Dowd et al., 2020). Papers in this category wondered to what extent demographic structures did condition the transmission dynamics. Overall, 13 papers (whose 5 from the socioeconomic category) found relevant spatial association between COVID-19 incidence rate, hospitalization rate or case-fatality rate and age groups.

Case-fatality rate (CFR) is the most common age-specific indicator disaggregated by cohort (Dudel et al., 2020). It can suffer from some critical underestimations (deaths outside hospitals, real number of asymptomatic confirmed cases), but its reliable estimation is crucial. During the early stage of the pandemic, its crude estimation arose some misunderstandings. In March 2020 apparent CFR in China showed to be 0.4% for 40–49 age interval, but it peaked 14.8% in patients older than 80 years. In Italy, those values were even worse (0.7% and 27.7%) with almost 97% of deaths occurring to patients older than 60 years (data quoted by Beam Dowd et al., 2020), but they were conditioned by the huge number of undetected cases.

Goldstein and Lee (2020, WP) suggested to define a CFR disaggregated not only by age but also by cause, to circumvent the heterogeneity of counts; thus, they could enhance the strong age-dependency of illness outcomes, with a death risk manifold higher in the elders. Levin’s et al. (2020) systematic review found a solid exponential relation between age and COVID-19 CFR, that ranges from 0.01% at 25 years to 4.6% at 75 years, and 15% at 85 years. Geographically, many authors agreed that almost the whole observed CFR variation among countries and regions mirrored their differences in age structure and in age-related exposition (Beam Dowd et al., 2020; Dudel et al., 2020; Levin et al., 2020). These interpretative corrections allowed to resize the exceptionality of the Italian case. The intensity in intergenerational contacts (Beam Dowd et al., 2020), was also indicated as able to shorten the “network distances” between the first imported cases and the elders, who would not be directly exposed per se to the mobility fluxes suspected to have spread COVID-19 (such as meetings, business travels; Bontempi et al., 2020).

By controlling this interference, the age structure of the first cases may predict which population cohort will be first hit, and that could explain the different evolution of the first pandemic stage in Germany, Italy and South Korea, whose starting outbreaks occurred in very different age cohorts. The age of the first COVID-19 cases could have even affected the reaction of the public authority, pushing it to underestimate the risk whereas first data came from less susceptible cohorts (Beam Dowd et al., 2020). This is what occurred in the UK, for which Verhagen et al. (2020) proposed to diversify the map of hospital saturation risk by the higher percentage of elders in some areas (Wales, Cornwall, Northumberland and Suffolk, the London neighborhood of Harrow).

Some studies coupled the GIS methods with traditional retrospective analyses: Dagnino et al. (2020, Brasil, NPR) found that the incidence peak in Rio Grande do Sul occurred in the 30–69 years cohorts; Zambrano et al. (2020, Honduras, NPR), noticed that almost 60% of confirmed cases were 60–79 years old, while the least affected cohorts were younger than 10 years (< 5%).

A core of demographic studies was related to the ethnic decomposition of the pandemic risk, typically in the United States. In New York City, Wadhera et al. (2020) observed that the Bronx, that has the highest percentage of minorities, people in deprivation and the lowest degree of education, had also the highest COVID-19 hospitalization and death rate. On the contrary, these rates were consistently lower in the wealthier Manhattan and Staten Island areas, mainly inhabited by whites, albeit their higher percentage of elderly. In Chicago, Kim and Bostwick (2020) observed that a higher percentage of African-American inhabitants was associated with higher pandemic risk levels, and that minorities were overrepresented in COVID-19 death rates. As already noticed in influenza epidemiology (Hutchins et al., 2009), it should be highlighted that the “ethnic variable” is but a proxy of deteriorated conditions stemming from long-term discriminations, and that it mediates other risk factors like economic status, household structure, chronic diseases, jobs in essential services (Ramirez and Lee, 2020). That could explain why covariance between COVID-19 and ethnicity appeared inconclusive in the geographical regression proposed by Mollalo et al. (2020), who found, indeed, a neat predominance of income inequalities.

#### Studies on climatic and environmental cofactors

The research question in these studies was whether (and how much) environmental variables could condition the virus transmission, and whether geographical patterns could be found and explained.

Within the medical community, the cyclization of the SARS-CoV-2 aggressiveness was strongly expected across all the communities in the temperate latitudes (Neher et al., 2020). It is known that coronaviruses have a typical seasonal trend, with a characteristic diffusion peak each year from December to March in the northern hemisphere (Neher et al., 2020). Influenza seasonality is generally explained (Mathews et al., 2009) through the cyclical weakening of the herd immunity induced by the previous seasonal flu.

A mediate correlation between the higher flu transmissibility and the reduction of air temperature seems also well-established, whereas the real correlation might be with the higher percentage of time spent indoor, in spaces where viral airborne concentration can maximize, and interpersonal distance is minimal (Mathews et al., 2009). This “correlation leap”, that is still not solidly ascertained in the medical literature, caused difficulties.

What geographers cared about the most was to understand whether the anisotropic propagation of the pandemic could be climate dependent. Researchers (Sajadi et al., 2020, NPR; Araújo and Naimi, 2020, NPR) could not avoid noticing that the pandemic had a clear preferential development along the mid-latitudes, like in Europe and North America, rather than getting rooted within other territories that are geographically and statistically better connected with China (like South-East Asia). A recurring conclusion was that this pattern was determined by the virus need of fresh and dry climate conditions to successfully complete its airborne phase.

Many of the surveyed analyses found, in fact, a significant negative correlation between COVID-19 incidence and temperature and humidity (Sajadi et al., 2020, NPR; Wang et al., 2020, NPR; Oto-Peralías, 2020, NPR; Araújo and Naimi, 2020, NPR; Paez et al., 2020; Runkle et al., 2020). This conclusion was the most recurring and was in line with the expected seasonality. Nevertheless, there were studies that found positive correlation between infections and humidity (Pirouz et al., 2020), positive correlation with temperature (Ma et al., 2020; Bashir et al., 2020), and no climatic correlations at all (Gupta et al., 2020). On the whole, 14 papers (≈33%) out of 43 papers constituting this thematic category found a negative correlation between COVID-19 propagation and atmospheric temperature, while 13 papers (≈30%) found negative correlation with atmospheric humidity. 5 papers (≈12%) found positive correlation with atmospheric temperature, 5 papers found positive correlation with atmospheric humidity, 8 papers (≈19%) found inconclusive results respect to temperature, 5 papers found no correlation respect to humidity, and 4 papers found no correlation with precipitation. 8 papers (≈19%) proved the relevance of latitude in the pandemic spreading, thus alluding to the expected seasonal forcing of viral infectious diseases. Significantly, 9 papers (≈21%) established the existence of a non-linear relationship between the COVID-19 incidence and climatic variables, or explicitly invoked climatic suitability ranges with upper and lower limits affecting the non-monotone trends.

Insofar, the general conclusion was that some form of correlation does exist, but it is challenging to be clarified, and some authors (Pirouz et al., 2020) acknowledged the existence of unknown patterns between the pandemic and climate, or patterns that are too ambivalent and non-conclusive to be generalized (Runkle et al., 2020; Zhang et al., 2020). Indeed, some researchers underlined the incomparably greater weight of other covariates, like population density (Gupta, et al., 2020). As for the geomorphological covariates, relevant patterns were not found, except for a weak association with altitude (Xiong et al., 2020; Gupta et al., 2020).

In the first part of 2020 the researchers had the urgency to understand if the boreal summer would slow down the exponential growth of cases without keeping the mitigation measures: that was disproved (Wang et al., 2020, NPR; Oto-Peralías et al., 2020, NPR). The covariance of ultraviolet radiation was also investigated, to bypass the mediate temperature-infection correlation, but results were non-conclusive (Gupta et al., 2020; Runkle et al., 2020).

Environmental analyses, in summary, encountered strong hindrances in converging towards general conclusions. The first one was that the global virus transmission could have easily concealed the weak climate-dependent signal. SARS-CoV-2 found an entirely available planet, with a ubiquitous and 100% susceptible host population, an ecological privilege not deemed to exist for the other seasonal coronaviruses (Baker et al., 2020). Thus, geographically, the pandemic beginning could develop very differently from interpandemic phases. Secondly, the different reactions of the public authorities could have strongly altered the distribution of cases, namely by how much they decided (or managed) to invest in diagnostic activities. This, indeed, exasperated the problem of undetected cases. Thirdly, many covariates might allegedly exist whose weight was disproportionally higher than the climate factors in fostering COVID-19 propagation, among which there is the “host’s behavior” (O’Reilly et al., 2020; Wang et al., 2020, NPR). This point is crucial, because, in many communities, indoor infection could be neatly preponderant (Araújo and Naimi, 2020, NPR), as witnessed by superspreading events and by the ambiguity of the findings about the infection rates in intertropical cities, where a positive correlation between infections and precipitation (Falcao Sobral et al., 2020) or temperature (Auler et al., 2020) is occasionally found. Finally, the biases induced by the preferred statistical methods could be so strong to affect the results (Briz-Redòn 2020, NPR). Therefore, current findings of spatial analyses about climate correlations dictate to be cautious, and to consider them as circumstantial.

#### Pollution studies

In late February 2020, the pandemic began its rapid community spreading in Europe, and the most harmful outbreaks first occurred in the middle of Po Plain, (Northern Italy), where, in the last week of March, apparent CFR reached 12% (Conticini et al., 2020), an alarming level if compared with the rest of Italy and the globally expected CFR. While the researchers were discussing about the CFR age-decomposition, other authors parallelly tried to find associations between the anisotropic progression of the pandemic and the different levels of exposure to pollutants experimented by human communities.

Northern Italy often shows some of the worst values of Air Quality Index (AQI) in Western Europe.^1^ This indicator, albeit not uniformly gauged in different countries, is mainly based on the ground air concentrations of PM_10_, PM_2.5_, O_3_, SO_2_ and NO_2_. They were the most studied compounds in the surveyed spatial analyses. A first exploratory study (Setti et al., 2020, WP) suggested that atmospheric particulate could operate as a physical carrier apt to ease the viral transmission by prolonging virus permanency in air suspension. It hypothesized also that an excessive number of days with PM_10_ and PM_2.5_ above the legal limits could become a boosting factor.

This interpretation encountered some perplexities because other almost coeval studies (Mollalo et al., 2020) found no correlation between incidence rate and pollution, or highlighted how, from a global comparison, this nexus should not be automatically taken for granted (Bontempi et al., 2020).

In medical literature, pollution is a notorious factor of prolonged inflammation and impairment of the respiratory tract (Conticini et al., 2020). Long-term exposure to PM_2.5_ e PM_10_ is known to lead to immune system overactivation, (Conticini et al., 2020), therefore, an individual living in a geographical area with high pollution levels is more likely to develop chronic respiratory diseases. Moreover, the long-term exposure to pollutants tends to maintain a high chronic inflammatory stimulus in the organism, also in young and healthy individuals. Altogether, 8 papers (which means ≈ 90% of this subset of studies) found positive association between at least one pollutant atmospheric compound and COVID-19 spatiotemporal increase. The most investigated compounds were PM_2.5_ (5) and PM_10_ (3), but also NO_2_, SO_2_ and O_3_ were considered for association.

On this basis, Coccia, (2020) found correlation between the number of days with PM_10_ excesses and infections and concluded that, in the first pandemic stage, pollution could predict the community transmission even better than social contacts. Murgante et al. (2020) observed a relevant spatial correlation between the worst pollution-affected and the worst COVID-19-affected Italian provinces.

These conclusions were consistent also in other countries like Peru (Badillo-Rivera et al., 2020, NPR) and China (Yao et al., 2020). Wu et al. (2020), studying more than 3000 U.S. counties, concluded that 1 μg/m^3^ increase in PM_2.5_ concentration was associated with 8% increase in COVID-19 CFR. Likewise, Xu et al. (2020), studying 33 Chinese cities, found significative correlation between AQI values and confirmed cases. Zhang et al. (2020) cautioned, however, that reciprocal feedbacks between pollution and meteorological variables may trigger complex nonlinear effects, with even opposite outcomes, and the direction of correlation was not always clearly explainable (Bashir et al., 2020).

A second subset of these studies investigated the “pollution covariate” as a dependent variable and assessed how it changed because of the pandemic. The multitemporal comparison of satellite images led the researchers to the unanimous constatation that, for some pollutants (above all NO_2_), the atmospheric concentration visibly dropped during national lockdowns (Bao and Zhang, 2020; Kanniah et al., 2020). This elicited many reflections about the extraordinary almost real-time capability of humans to modify the atmospheric composition, but some pernicious feedbacks did not go unnoticed, like the abrupt increase of O_3_, made possible by its trade-off with nitrogenous compounds (Collivignardelli et al., 2020). Overall, 8 papers found NO_2_ atmospheric reduction during the lockdown; 6 papers found PM_2.5_ reduction, 4 papers PM_10_ reduction, 5 papers CO reduction, 4 papers SO_2_ reduction, 4 papers O_3_ increase.

#### Mobility analyses

SARS-CoV-2 manifested high aggressiveness in its quick spreading among local communities, leaping from its circulation throughout the global business networks to the thinnest contact networks of the most remote villages, in just few weeks. This behavior prompted many authors in mapping its possible access routes. Hence, their research question was: “how did the pandemic take advantage of human mobility to expand worldwide?”

On the whole, 9 (≈24%) of the 38 mobility analyses worked on international flight fluxes to evaluate quantitatively the risk of importation of confirmed cases from abroad. 5 papers (≈13%) relied upon the analysis of visits/contacts with the primary outbreak of Wuhan finding positive correlation between the number of these connections and the secondary outbreak entity. 4 papers (≈11%) mapped the possible role of local transport infrastructures (roadways, seaports, airports) in fostering the pandemic propagation. 6 papers (≈16%) considered contact-tracing measures at the individual level. More widely, 17 papers (≈45%) adopted data mining on big data repositories to detect aggregate mobility flows. 10 out of them extracted their data from social networks or data aggregators (Google, Baidu, Tencent, Facebook). Travel bans were the focus of 14 papers (≈37%), while, in general, 9 papers (≈24%) adopted a “scenario” study design to carry out prospective analyses and forecasting.

Mobility studies in China focused upon the massive relocations that took place in connection with the Lunar New Year holydays. The recurring methodology was based on the analysis of the mobile phone big data (through Baidu, Tencent), and their combination with transportation companies’ data. Zhou et al., (2020) found the maximum pandemic risk in those areas, like the Shanghai-Hangzhou and Canton-Shenzhen megacities, that received intense inward fluxes of workers coming back from their vacation. Another investigation strategy was the modeling of outward flows from Wuhan’s primary outbreak, with the aim to identify the relative frequency and the geographical distribution of the confirmed cases stemming from secondary outbreaks.

Shared conclusions were that the volume of travelers leaving Wuhan was strongly correlated with confirmed cases in the destination provinces or prefectures, and, conversely, the number of cumulative confirmed cases was strongly correlated with the total flow of arrivals from Wuhan. Thus, the variable “arrivals from the primary outbreak” was an excellent predictor of secondary outbreaks’ entity outside Hubei (Jia et al., 2020). Exported cases from Wuhan before the quarantine triggered local transmission chains in different provinces, both adjacent and distant (Henan, Guangdong, Zhejiang), but, once the *cordon sanitaire* was settled, this correlation faded, and new locally expressed factors took over (Chow et al., 2020), which indirectly documented the efficacity of quarantine in halting mobility fluxes (Jia et al., 2020).

The researchers soon turned their attention on international mobility because Chinese pre-lockdown clusters were open routes for the virus’ planetary propagation (Chow et al., 2020). Big data generated by flight data aggregators (IATA, SABRE) were mostly employed to build risk cartographies by merging data about travelers exiting China with destinations’ vulnerability indexes. However, Christidis and Christodoulou (2020) highlighted the unexpected weakness of mobility correlations: once the pandemic made its access to global transportation networks, stochastic fluctuations seemed to be more relevant for the outcome than the network structure itself.

Many analyses used mobility to assess the overall effect of mitigation measures: Chinazzi et al. (2020) concluded that quarantining Hubei delayed global pandemic progression by 3–5 days in China and much more abroad, where imported cases were reduced by 80% since mid-February. de Oliveira et al., (2020, NPR) shared similar findings: they compared ten countries in South and Central America and found that only the countries that imposed an official lockdown were able to flatten the epidemic curve. Besides, Jarynowski et al., (2020, Poland, PP) found that local workers’ mobility could explain most of the variance of the pandemic dynamics across Polish districts, while workers’ mobility abroad operated as a statistical accelerator for the virus importation. Brazilian geographers noticed how air, motorway, and fluvial displacement facilities showed strong association with the territorial propagation of the pandemic, and indeed allowed the virus to carry out the “scale ratio leap” from global to local networks (Aleixo et al., 2020).

Through this *excursus,* we were able to deepen the rich production of epidemiological mapping and spatial analyses of COVID-19. Hence, we drafted a balance about the main sub-fields of investigation, the most followed approaches, and the possible points of friction.

### A general routine for COVID-19 epidemiological mapping and spatial analysis

**Fig. 7:**
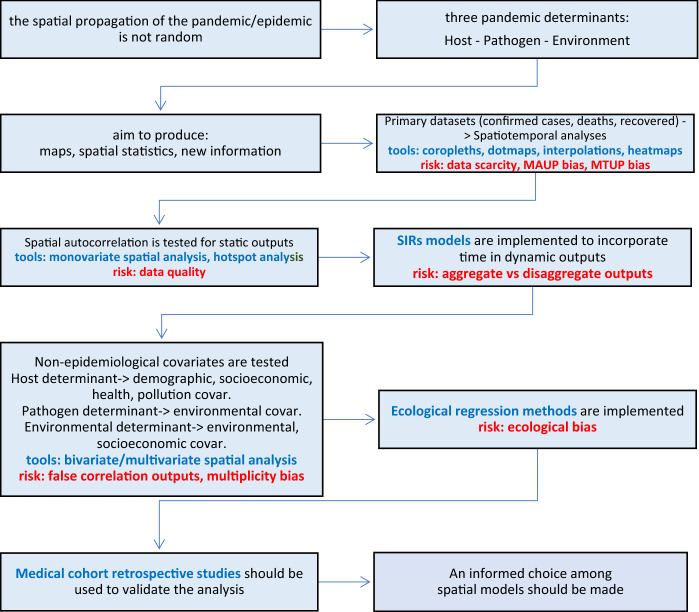
The workflow summarizes and condenses the possible steps (in black), the available instruments (in blue) and the critical points (in red) of a generic epidemiological-geographical study, on the basis of the outputs of this survey

Following the screening and the analysis of the existing research, a tentative (problematized) eight points routine for a “COVID-19 epidemiological-geographical study” could be drawn out from the surveyed manuscripts (Fig. 7).

1. In every case the first assumption is that the pandemic spatial diffusion is not randomly determined. The basic principles of epidemiology are that three global Determinants are required for an epidemic (or pandemic) to occur (Desenclos and de Valk, 2005; Che, 2019): the new or existing *Pathogen* attain characteristics able to trigger its exponential replication; the *Host* presents suitable characteristics and favorable behaviors apt to sustain the replication of the pathogen; the *Environment* (sensu Eisenberg et al., 2007) guarantees prolonged idoneous conditions for the spreading of the pathogen.
2. Secondarily, in most cases a geography-based method is applied with the general purpose to produce a map or a spatially-based statistic output and the specific purpose to generate new information. A first severe hindrance occurs: data are tendentially scarce, and the very first choice is to rely solely upon “*stricto-*sensu” epidemiological data (confirmed cases, deaths, hospitalizations). This is the case of spatiotemporal analyses: simple synchronic or diachronic maps were obtained, but they proved to be quite effective in generalizing (see paragraph 3.2.1). For these results, vector analysis in GIS environment appeared to be the best and the most immediate methodology (coropleth maps, temporal overlay, IDW, kriging and other interpolations, heatmaps) used by the researchers to produce the primary explorative mapping of affected territories.
3. Spatial autocorrelation of epidemiological data can inform more conclusions, but researchers are required to rely on strong and updated knowledge of the territory to draw out more well-grounded observations about the pandemic behavior. In this case some well-known geostatistical algorithms were massively adopted (monovariate Moran’s I, Getis-Ord G*, Kulldorff spatial scan statistic) and hotspot analysis was successfully addressed to detect and hierarchize outbreaks (Desjardins et al., 2020; Ramirez and Lee, 2020).
4. Refined conclusions can be attained by strengthening the treatment of time as the main geographical variable: here the link with classic epidemiology is robust and relies upon the implementation of the compartment models like SIR, which however implies that the scientific community has already reached consensus upon a realistic value of the basic replication number. Again, in that case, the previous knowledge of the territory and the studied communities and the ability to implement disaggregated solutions is crucial to let the researchers elaborate new spatially related findings, and results may be notably consistent and innovative (Kergaßner et al., 2020; Scarpone et al., 2020; Bizzarri et al., 2020).
5. Another step is to add non-epidemiological covariates to the study. Demographic covariates are easily available. In this case (paragraph 3.2.4), following the traditional epidemiological theory, the researchers address the Host Determinant in term of population entity, age, and distribution. Health covariates (paragraph 3.2.3) are less easily available, but they can manifest strong relations: through them, the researchers, again, address the Host Determinant (in term of organism susceptibility to the infection or in term of probability to receive adequate treatment). Socioeconomical covariates are, instead, utterly uneven. With them, the Host Determinant is addressed more ambiguously (Does income affects health? Does built environment condition gatherings? Does accessibility to services affect lifestyle?). Covariates can be spatially managed with spatial bivariate or multivariate correlation; in this case, besides the traditional Pearson, Kendall, Spearman and other tests, also Anselin’s LISA method, geographically weighted regressions, and Getis-Ord statistics were frequently applied. Environmental covariates are instead more ephemeral, and their spatial analysis gave more erratic results (paragraph 3.2.5). They are used to address both the Environment and the Pathogen Determinant. Anyway, for a new disease the second is originally unknown, and only the first one can be parametrized, but it is made of territorially continuous variables not inherently attached to the host. The contribute of temperature, humidity, precipitation, and climate to the spreading can be excessively smoothed respect to other stronger host-related cofactors. Moreover, in many cases, the climatic spatial analyses, hurried by the emergency, were forced to define “static” study designs and drafted only partial snapshots of the epidemiological situation, biased by the fact that a 100% susceptible host population acted as the main driver of the pandemic (Carlson et al., 2020a). Pollution covariates might appear, in their turn, as an exception, as they are virtually “incorporable” within the host as health conditions (paragraph 3.2.6). This probably makes the outputs of their correlation studies slightly more coherent. Nonetheless, no conclusion should be pointed out without prior ascertainment of the medical effects of the individual long-term exposure to pollution. Mobility is one of the nearest covariates to spatiotemporal analysis (see Paragraph 3.2.7), and it addresses the Host Determinant in term of host’s behavior. Big data were used to evaluate the effect of travel bans and proved how mitigation can be attained at least acting on human displacements. In our opinion, this type of datasets should be investigated more granularly respect to the geographical pattern of the pandemic propagation, above all in the onset period, whereas the disaggregation between imported and local cases is still feasible, and containment measures might be still implemented. Mobility appeared to be the most promising variable, but it is still the least easily manageable, because it appears to be affected by stochastic dynamics that are difficult to be modeled (Christidis and Christodoulou, 2020) and because an excessive disaggregation of mobility data may entail data confidentiality issues (Zhou et al., 2020).
6. As already mentioned, medical cohort retrospective studies should always inform a spatial analysis to corroborate its conclusions or to give a well-grounded basis for the spatial generalizations, as long as their results become available. In that way, the mapping of covariances can proficiently intervene in the epidemiological study to generate, with GIS, new knowledge in terms of new “reasonably justified inferences”. That is probably the sole possibility to incorporate the “raster” suspected covariate (built environment, pollution, climate range) or the “metonymic” suspected covariate (income, job, chronic conditions, provenance, historical exposure) into the “vector point” epidemiological variable (the confirmed case, the death, the recovered, etc.) to have a better counterproof about the spatial covariance.
7. Ecological regression methods could represent another efficient escape rope in epidemiological geography, as suggested by Wu et al. (2020), because they can approximate the exposure story of the patient in the absence of individual clinical data. This kind of models suffers, however, from the ecological bias and prevent the researchers to directly derive conclusions about individual-level association, but adding retrospective cohort studies can moderate this risk.
8. That finally leads to the open question about the best method to spatially shape the COVID-19 dynamics. This question, indeed, is feeding an ongoing vigorous debate in biogeography. As SARS-CoV-2 is a new species of virus, some ecologists attempted to define its most probable areal of expansion through the species distribution models, which implies to apply the ecological regression on environmental variables (Araújo and Naimi, 2020, NPR). The proposal was criticized on the basis of the incomparably higher weight of the host’s behavior (human interactions) respect to the environmental suitability in letting the airborne phase of COVID-19 complete successfully (Carlson et al., 2020b; Gutiérrez-Hernández and García, 2021a). Indeed, both sides have strong arguments for their choice (Araújo et al., 2020). In fact, other authors noticed that traditional SIR methods are very efficient at the coarse level, but, when they are not spatially disaggregated or they have poor access to real data, they threaten to miss many granular dynamics that may turn out to be crucial (Bizzarri et al., 2020; Benedetti et al., 2020).

## Discussion

Along the first pandemic year, the researchers attained remarkable results by applying geographical approaches. We identified few core topics that elicited high inquiry concerns, and in which applied geography rapidly specialized, attaining, in some cases, converging conclusions. Some background elements seem to have emerged. Dependent variables (e.g., confirmed cases) and socioeconomical covariates appeared to be better to be managed, and their analysis produced consistent results, with some possibilities of generalization. Common spatiotemporal trends were that the pandemic had a first rapid stage, in which, by aggressing a given community as an exogenous disturbance, it attacked wealthy classes and areas, verisimilarly because of their higher global interconnectedness with foreign sources (tourism, job travels, study permits, pilgrimages). In a second stage the pandemic became epidemic and took advantage of preexisting weakness lines in human societies. Everywhere an abrupt shift of epicenters towards the most fragile and deprived social classes was noticed. The latter were, in the end, far more exposed to infections and suffered from their smaller material chances to exert self-protection (lower access to remote-work, poor physical space for social distancing, bad-quality information, no savings to face prolonged confinement, less customized cures, fewer protection devices). Therefore, the first signs of endemism were given by the rooting of long-period outbreaks within crowded and deprived urban areas (Bermudi et al., 2020, NPR; Bag et al., 2020). The demographic covariate pattern was quite clear as well: the pandemic arrived through young and subclinical individuals, and then manifested its worst effects on the elders, among which only the most affluent component had enough means to guarantee self-protection. The specific structure of national population pyramids informed the real case-fatality rate (Beam Dowd et al., 2020). Likewise, the outcome related to health covariates was quite precise: the pandemic quickly manifested its worst outputs in those cohorts already burdened with the chronic diseases typical of wealthy societies (asthma, obesity, hypertension, diabetes, heart conditions) and triggered the collapse of all the ordinary health systems that lacked conversion protocols or resources to implement them (Gross et al., 2020). However, whereas climatic and environmental independent variables were examined, such an unambiguous interpretation tended to fade, and some conclusions could even be perceived as contradictory. At this very point, the MAUP bias, i.e., the strong scale-sensitiveness of many spatial associations, and the MTUP bias, i.e., the variability of geographical data when aggregated in different timespans, fatally manifested themselves (Briz-Redon, 2020; Kwan, 2018). Indeed, they stem from the Achilles’ heel of epidemiological geography, namely the metonymical usage of territory to study population health issues. In that way, contrasting and counterintuitive observations were produced (Bashir et al., 2020; Ma et al., 2020; Paez et al., 2020) and the scale-dependency of spatial correlations emerged (Xiong et al., 2020). This might be unavoidable, for at least two structural factors. The first one is ingrained in the object of study and corresponds to the “host’s behavior” factor, namely the fact that the statistical reaction of humans to the variation of climate and environmental drivers can often outpace the impact that the latter exert upon the airborne phase of the viral cycle, or, also, the possibility that another covariate may trigger some form of positive or negative feedback (Zhang et al., 2020). The second factor is inherent to the research process and may be defined as “the multiplicity bias”. Recently, in fact, Gutiérrez-Hernández and García (2021b) pointed out how observational studies in COVID-19 correlation tests research risk to be affected by a much greater number of type I errors than what is generally believed. This should not discourage a pragmatic epidemiological geography in its effort to build up coherent and consistent overviews (Kundi, 2006). On the contrary, it urges geographers to keep constant awareness on the “host’s behavior” factor, to multiply statistical models and to strengthen the controls upon spatial covariates with the help of an interdisciplinary approach *stricto *sensu, as well as to implement tools to control type I error inflation. Through a proper study design, epidemiological geography is asked to engage with a permanent and critical confrontation with cohort (Shi et al., 2020) and laboratory (Baker et al., 2020) experimental data, so that a litmus test may always be available in the description of the nonlinear and multifactorial structure of reality.

## Conclusions, limitations and further developments

With this survey we hopefully offered a more painstaking grounding to the systematization of the geographical analyses of the pandemic, both by letting a possible study design routine emerge from the magmatic scientific production of 2020, and by emphasizing the major known weak points that geographers encountered in their task. A limit of this study is that the chosen thematic categories might be questionable. A future effort should be to engage the systematic review of each category. The sole fact that the pandemic is still ongoing, at the time we are writing, urges to perform a radical update of our findings as soon as possible. No language filters were applied on the paper retrieval, but a strong bias may be derived using keywords in English. The number of papers circulating in the second half of 2020 was likely to be underestimated, because paper indexing in search engines may require long time to become effective. Finally, it cannot be excluded that the number of papers not finding any relevant correlation among the covariates was underestimated because of the “publication bias” (Gutiérrez-Hernández, 2021b). Future research should consider that the geographers’ toolkit, constituted by GIS, statistical analysis and geostatistics may be fruitfully applied in spatial analysis of infectious diseases, but with the highest possible awareness about the fundaments of epidemiology. The classic theory about the epidemic/pandemic determinants (the Pathogen, the Host, the Environment) informs the entire reliability and robustness of COVID-19 studies, be they spatial or not. Epidemiological Geography stands between these three subjects—cartography, statistics, and epidemiology—and can borrow all their explanatory potential to produce high quality cartography. At the same time, it should carefully deal with the weak point of each one: the MAUP/MTUP bias in cartography, the impossibility of statistics to give self-explanatory causative links, and the and multicausal and non-linear origin of epidemic pulses. Through the framework we tried to draft, we hope that the geographers and spatial analysts interested in epidemiological maps could get some hints about the risks and the potentialities in approaching the mapping of emerging infectious diseases, and, in particular, two specific clues about forthcoming investigations. The first one is the methodological urgency to incorporate individual-level clinical data from retrospective studies in spatial correlations, to couple multivariate epidemiological cartography with field data, and to alleviate the three mentioned drawbacks. The second one is the general need to refine the geographical appraisal of correlations using the host’s behavior covariates, inasmuch as they are demonstrating to be the most relevant for the pandemic outcome, but also the less known and the most poorly modelized.

## Footnotes

1 https://airindex.eea.europa.eu/Map/AQI/Viewer/.
